# Mean platelet volume could be a promising biomarker to monitor dietary compliance in celiac disease

**DOI:** 10.3109/03009734.2011.581399

**Published:** 2011-06-29

**Authors:** Tugrul Purnak, Cumali Efe, Osman Yuksel, Yavuz Beyazit, Ersan Ozaslan, Emin Altiparmak

**Affiliations:** ^1^Ankara Numune Education and Research Hospital, Department of Gastroenterology, Ankara, Turkey; ^2^Hacettepe University Medical School, Department of Gastroenterology, Ankara, Turkey; ^3^Ankara Diskapi Education and Research Hospital, Department of Gastroenterology, Ankara, Turkey; ^4^Ankara Yuksek Ihtisas Education and Training Hospital, Department of Gastroenterology, Ankara, Turkey

**Keywords:** Celiac disease, dietary adherence, mean platelet volume

## Abstract

**Background:**

Celiac disease (CD) is an autoimmune disease that develops in patients with a genetic predisposition, incurring a susceptibility to gluten-containing foods such as barley, wheat, and rye. The elimination of gluten from the diet is the main therapeutic approach and usually leads to clinical and laboratory improvement. There are no ideal markers that objectively assess dietary compliance in CD patients.

**Materials and methods:**

Sixty newly diagnosed CD patients (male/female: 43/17) and 40 healthy subjects (male/female: 23/17) were enrolled in this study. The diagnosis of CD was established by both histological findings of duodenum biopsy (total villous atrophy and lymphocytic infiltration) and positive antibodies against endomysium or gliadin.

**Results:**

A significantly higher mean platelet volume (MPV) was observed in the CD group compared with healthy subjects (8.45 ± 0.96 fL versus 7.93 ± 0.63 fL; *p* = 0.004). After introduction of a gluten-free diet, the MPV of CD patients in the dietary adherent group was significantly lower than that of the non-adherent group (8.09 ± 0.6 fL versus 8.9 ± 1.08 fL; *p* = 0.001). Overall dietary adherence rate was 71.6% (43/60 CD patients). In the dietary compliant group, initiation of gluten-free diet was associated with a significant decrease in MPV from base-line values (8.56 fL versus 8.25 fL; *p* = 0.008). In the non-adherent group, MPV on 3-month follow-up was higher than at base-line (8.05 fL versus 8.91 fL; *p* = 0.001).

**Conclusion:**

MPV could be a promising and easily available biomarker for monitoring of dietary adherence in CD patients at a low cost in comparison with other modalities.

## Introduction

Celiac disease (CD) is an autoimmune disease which affects genetically predisposed individuals, making them susceptible to gluten, which is found in foods such as barley, wheat, and rye. Progressive inflammation of the small intestine is the predominant underlying pathology of the disease, eventually resulting in malabsorption if gluten-containing foods are not restricted (1). A correct diagnosis is usually based on the detection of serum antibodies against endomysium and gliadin followed by histopathological confirmation by a biopsy of the small intestine, using criteria described by Marsh (intraepithelial lymphocytes, villus atrophy, and crypt hyperplasia) (1–3). Elimination of gluten from the diet is the main therapeutic approach, resulting in significant clinical and laboratory improvements in the majority of CD patients. None of the markers available today are reliable indicators of dietary compliance in patients with CD. Moreover, a group of patients present with subtle clinical and laboratory abnormalities which may delay the diagnosis (4). The early recognition of CD in such patients is of great importance to prevent the development of CD-related complications (osteopenia/osteoporosis and vitamin and mineral deficiencies due to malabsorption), the most feared of which being the development of T-cell lymphoma (5).

Mean platelet volume (MPV) has recently been recognized as an inflammatory marker in various conditions including ulcerative colitis, acute pancreatitis, and myocardial infarction (6–8). While some studies demonstrated a negative correlation between MPV and inflammatory activity, other investigators have reported on an association between increased MPV and disease severity**.** The relationship between MPV and CD was first reported by O'Grady et al. (9). No study has so far reported MPV as a biomarker for monitoring of dietary adherence in CD patients over time. The purpose of the present study was to evaluate the impact of MPV as a monitor of disease severity in CD.

## Materials and methods

This study was carried out between March 2009 and June 2010 on 60 newly diagnosed CD patients admitted to the gastroenterology department of Ankara Numune Education and Research Hospital. A diagnosis of CD was established by positive antibodies against gliadin and/or endomysium and confirmed with histological findings of duodenum biopsy based on modified Marsh classification (9,10).

The control group consisted of patients who were diagnosed with functional gastrointestinal disorders, all of whom had a normal complete blood cell count (CBC), erythrocyte sedimentation rate (ESR), and serum C-reactive protein (CRP) levels, as well as being negative for antibodies against gliadin and endomysium. Written informed consent was obtained from all the participants in the study.

Patients with heart failure, peripheral vascular disease, acute or chronic infection, cancer, hematologic and hepatic disorders, and a history of drug use (non-steroid anti-inflammatory drugs, anticoagulant medications, and oral contraceptives) were excluded from the study.

All CD patients approached for inclusion into the study were subjected to ultrasonographic examination of the abdomen by an experienced operator using the General Electric LOGIQ P5 device. A 3–5 MHz (3.5 C) convex transducer probe (General Electric Healthcare, Milwaukee, WI) was used for the measurements. The spleen size was evaluated in the right lateral decubitus position. Measurements were obtained of the splenic length with the maximum dimension during suspended respiration. Splenic lengths of 80–130 mm were considered as normal, and patients with splenic atrophy were excluded from the study.

The implications of a diagnosis of CD were discussed at length with all patients, after which the necessary gluten-free dietary recommendations were given, with particular stress on the importance of adherence.

A follow-up visit was scheduled for 3 months later for each patient, when participants were asked to complete a standard questionnaire regarding their adherence to a gluten-free diet. Subjects were subsequently divided into two groups based on their responses to questions on the questionnaire: Group 1 consisted of patients deemed adherent to a strict gluten-free diet, whereas those with no or intermittent adherence to the diet were placed in Group 2.

All CBC analyses were performed in the hematology laboratory of our hospital. All CBC analyses both on admission and at control were performed within 2 hours of blood sample collection with the use of a Beckman Coulter (High Wycombe, UK) Gen-S automated analyzer.

The Statistical Package for Social Sciences (SPSS) for Windows v. 17 was used for all statistical analyses. All data were given as mean values plus/minus SD. For continuous variables, the Mann–Whitney *U* test was used to analyze the variance among groups. Chi-square test was used for comparison of categorical variables. A *p*-value of 0.05 and below was considered statistically significant.

## Results

**Figure 1.**
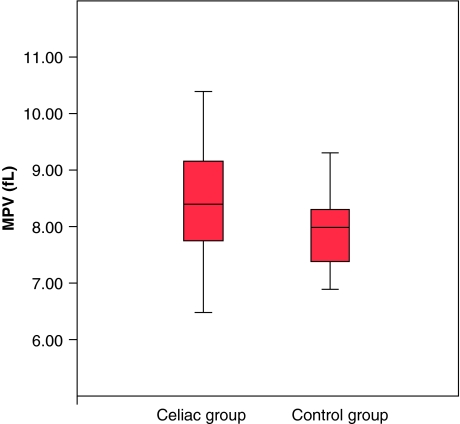
Comparison of MPV values between patients and healthy controls on admission.

Sixty CD patients (43 males, 17 females) and 40 healthy controls (23 males, 17 females) were enrolled during the study period. Demographic and clinical characteristics of all participants are summarized in Table I. Mean age, gender distribution, and smoking habits were similar in both groups. MPV in initial diagnosis was significantly higher in the CD group compared to healthy controls (8.45 ± 0.96 fL versus 7.93 ± 0.63 fL; *p* = 0.004) (Figure 1). Similarly, patients in the CD group had a higher mean platelet count compared to their healthy counterparts (346 × 10^9^/L versus 264 × 10^9^/L; *p* = 0.001). Laboratory values of the study population are summarized in Table II.

**Table I.** Demographic features of the patients and controls.

	Patients (*n* = 60)	Controls (*n* = 40)	*p*
Age (years)	35.3 ± 12.4	40.1 ± 12.5	0.61
Gender F/M	43 (71.7%)/17 (28.3%)	23 (57.5%)/17(42.5)	0.143
Smoking +/-	20 (33.3%)/40 (66.7%)	12 (30%)/28 (70%)	0.94
Spleen size (mm)	92 ± 12	93 ± 11	0.86
Dietary adherence	47/60 (71.6%)		

**Table II.** MPV and other inflammatory markers in patients and controls.

	Patients (*n* = 60)	Controls (*n* = 40)	*p*
MPV (fL)	8.45 ± 0.96	7.93 ± 0.61	0.004
Platelet count (× 10^9^ /L)	346 ± 121	264 ± 71	0.001
CRP (mg/L)	3.05 ± 1.22	3.1 ± 1.0	0.148
ESR (mm/h)	17.7 ± 17.4	16.4 ± 11.7	0.156
WBC count (× 10^9^ /L)	7.0 ± 1.72	7.5 ± 2.01	0.245

CRP = C-reactive protein; ESR = erythrocyte sedimentation rate; MPV = mean platelet volume; WBC = white blood cell.

Based on results of the questionnaire at the follow-up visit at the third month, 43 of 60 (71.6%) CD patients adhered to a strict gluten-free diet, compared to 17 patients (29.4%) who were deemed non-adherent. MPV of the adherent group was significantly lower than that of the non-adherent group (8.09 ± 0.6 fL versus 8.9 ± 1.08 fL; *p* = 0.001). In the dietary compliant group, introduction of a gluten-free diet resulted in a significant decrease in MPV compared to base-line (8.56 fL versus 8.25 fL; *p* = 0.008). In the non-adherent group, MPV at 3-month follow-up was higher than the value at base-line (8.05 fL versus 8.91 fL; *p* = 0.001) .

## Discussion

Our findings indicate that MPV was augmented in patients with newly diagnosed CD compared to healthy controls. Among CD patients, mean MPV values showed a tendency towards normalization over time in patients in the diet-adherent group. Conversely, in non-adherent patients and the patients with intermittent transgressions, the mean MPV value continued to increase from the starting value.

CD is a chronic inflammatory disorder and requires life-long treatment and follow-up. Despite our understanding of new aspects of the disease, treatment remains unchanged and constitutes elimination of gluten from the diet (11). Dietary compliance is generally based on patient self-reports, and there are very few objective criteria to evaluate the dietary compliance among CD patients. Although demonstration of histological improvement is the generally accepted gold standard, this remains an invasive and impractical method for the routine follow-up of a selected group of patients (12). Until now, a reliable, non-invasive measure of dietary adherence has not been described. Follow-up of antibody titers, particularly against tissue transglutaminase (Ttg), endomysium, and gliadin, has been proposed as a good indicator of transgressions in CD patients. However, there are some limitations in routine clinical practice (13,14). For example, certain disorders (autoimmune hepatitis, giardiasis, refractory CD) may result in persistently high antibody titers, making the interpretation of results quite difficult (5). Furthermore, antibodies against endomysium and Ttg may not be helpful in detecting minor or intermittent transgressions in CD patients. On a similar note, some patients may have extremely high base-line antibody titers on initial diagnosis, and a delayed return to normal levels in such cases may mislead clinicians (5,15).

Many hematology analyzers have included MPV measurement in their repertoire, giving some information about platelet activation and function. In recent years, some studies have investigated a possible association between MPV and several inflammatory conditions such as myocardial infarction, stroke, diabetes, ulcerative colitis, chronic hepatitis B, and acute pancreatitis (6,8,16,17). Yuksel et al.(6) reported low levels of MPV to be related to more severe activity in ulcerative colitis patients, postulating that MPV may be equal to other conventional markers as an indicator of disease severity. Conversely, in a study by Klovaite et al. (18) increased MPV was found to be an independent risk factor for myocardial infarction. Based on these conflicting reports, it would seem that both high and low MPV have a diagnostic and prognostic value for different inflammatory conditions.

Our study results are consistent with findings reported by O'Grady et al.(19), where the MPV values of three groups (CD patients, normal controls, and splenectomized patients) were compared. The authors discovered that CD patients with intact spleens had higher MPV values and platelet counts. However, these investigators did not evaluate the effect of a gluten-free diet on MPV.

The high MPV values observed in our study group of newly diagnosed CD patients may be a reflection of on-going intestinal inflammation; while on the other hand, normalization of MPV values with the introduction of a gluten-free diet may indicate resolution of intestinal inflammation.

Our data suggest that MPV could be a promising and easily available biomarker for monitoring the dietary adherence of CD patients at a low cost in comparison with other modalities.
